# The assessment of the serum levels of TWEAK and prostaglandin F2α in COVID – 19

**DOI:** 10.3906/sag-2006-96

**Published:** 2020-12-17

**Authors:** Demet YALÇIN KEHRİBAR, Mustafa CİHANGİROĞLU, Emine SEHMEN, Bahattin AVCI, Mustafa ÇAPRAZ, Maruf BORAN, Caner GÜNAYDIN, Metin ÖZGEN

**Affiliations:** 1 Department of Internal Medicine, Faculty of Medicine, Ondokuz Mayıs University, Samsun Turkey; 2 Department of Infection Diseases and Clinical Microbiology, Faculty of Medicine, Amasya University, Amasya Turkey; 3 Department of Infection Diseases, Gazi State Hospital, Samsun Turkey; 4 Department of Medical Biochemistry, Faculty of Medicine, Ondokuz Mayıs University, Samsun Turkey; 5 Department of Internal Medicine, Faculty of Medicine, Amasya University, Amasya Turkey; 6 Department of Intensive Care Unit, Faculty of Medicine, Amasya University, Amasya Turkey; 7 Department of Pharmacology, Faculty of Medicine, Ondokuz Mayıs University, Samsun Turkey; 8 Department of Rheumatology, Faculty of Medicine, Ondokuz Mayıs University, Samsun Turkey

**Keywords:** COVID-19, TWEAK, leukotrienes, inflammation, prostaglandin

## Abstract

**Background/aim:**

It is claimed that aberrant immune response has a more important role than the cytopathic effect of the virus in the morbidity and mortality of the coronavirus disease 2019 (COVID-19). We aimed to investigate the possible roles of tumor necrosis factor-like weak inducer of apoptosis (TWEAK)/Fn14 pathway and leukotrienes (LT) in uncontrolled immune response that occurs in severe acute respiratory syndrome coronavirus 2 (SARS-CoV-2).

**Materials and methods:**

This study included 25 asymptomatic patients and 35 patients with lung involvement who were diagnosed with COVID-19 as well as 22 healthy volunteers. Lung involvement was determined using computed-tomography. Serum TWEAK, LTE4, and prostaglandin F2α (PGF2α) levels were determined.

**Results:**

Compared with the healthy control group, TWEAK, LTE4, and PGF2α levels were higher in the group of SARS-CoV-2 infection without lung involvement. In the group of SARS-CoV-2 infection with lung involvement, age, fibrinogen, sedimentation, C-reactive protein and ferritin, TWEAK, LTE4, and PGF2α levels were higher, and lymphocyte levels were lower compared with the asymptomatic group.

**Conclusions:**

In the study, TWEAK and LTE4 levels increased in cases with COVID-19. These results support that TWEAK/Fn14 pathway and LT may involved in the pathology of aberrant immune response against SARS-CoV-2. Inhibition of each of these pathways may be a potential target in the treatment of COVID-19.

## 1. Introduction

Severe acute respiratory syndrome coronavirus 2 (SARS-CoV-2), which emerged in December 2019 and is a new member of the coronavirus family, infected millions of people and caused thousands of deaths worldwide. There is not a vaccine, and deaths continue to occur at the same pace. Many epidemiological studies conducted so far have revealed considerable amount of data on the transmission route and clinical presentation of the disease. Thus, methods of protection from SARS-CoV-2 and protocols for identifying possible cases have been established.

It has been noted that the main cause of death associated with SARS-CoV is a cytokine storm [1]. Although cytopathic effects of the virus are believed to be noteworthy in the severity of the disease, experience and clinical results from other coronaviruses, severe acute respiratory syndrome coronavirus (SARS-CoV-2), and Middle East respiratory syndrome virus (MERS-CoV) indicate that the aberrant host immune response causes an inflammatory cytokine storm and mortality [1]. Similar to the inflammatory cytokines in SARS and MERS, patients with SARS-CoV-2 also have increased plasma concentrations of inflammatory cytokines, such as interleukins (IL)-6 and IL-10, tumor necrosis factor-α (TNFα), granulocyte colony-stimulating factor (G-CSF) [2]. The effectiveness of the IL-6 inhibitor tocilizumab, which is used to stop cytokine storm, supports this view [3].

TNF-like weak inducer of apoptosis (TWEAK) is a member of the TNF ligand family and is first synthesized as a transmembrane protein of 249 amino acids [4]. Although it was initially described as an apoptosis stimulant [5], subsequent studies have shown that it is involved in many inflammatory and immunological processes [6,7].

TWEAK binds to fibroblast growth factor-inducible 14 (Fn14), its only known receptor, [8], and it stimulates the release of cytokines such as TNFα, IL-1, IL-6, G-CSF, and interferon-γ monocyte chemoattractant protein 1, macrophage inflammatory protein 1 alpha, intercellular adhesion molecule 1, vascular cell adhesion molecule 1 (VCAM-1), and interferon-γ-induced protein 10 (IP-10) from TWEAK tissues that increase with inflammation [9-11]. These data show that the TWEAK/Fn14 pathway makes a considerable contribution to the inflammation occurring in the tissues, and the excessive or persistent upregulation of this pathway plays an important role in the pathogenesis of some pathological inflammatory diseases such as systemic lupus erythematosus and rheumatoid arthritis (RA) [12-14].

Leukotrienes (LT) are generated as a result of the arachidonic acid metabolism and are lipid mediators of the inflammatory response. LTs are divided into two as di-hydroxy acid LT (LTB4) and cysteinyl LTs (CysLTs; LTC4, LTD4, and LTE4). LTE4, which is one of the CysLTs, is the form that is more stable and abundant in biological fluids compared with others. CysLTs is known to play an important role in inflammatory diseases of the respiratory tract, such as asthma [15], pulmonary inflammation and fibrosis [16], and acute respiratory distress syndrome (ARDS) [17-19].

It is considered that the more morbid and mortal course of COVID-19 in some individuals is not due to the cytopathic effect of the virus but rather due to the aberrant immune response developed by the host against the virus [3]. In this study, we aimed to investigate the possible roles of TWEAK/Fn14 pathway and LTs in the immune response caused by SARS-CoV-2.

## 2. Materials and methods

### 2.1. Patients

This study included patients who presented from March 30 to April 30, 2020, with a clinical presentation leading to suspicion of SARS-CoV-2 infection and were diagnosed with SARS-CoV-2 infection using polymerase chain reaction (PCR) of swab samples.

Demographic, clinical, laboratory, and radiological data of all patients were recorded. Based on computed tomography findings, patients with SARS-CoV-2 infection were divided into two subgroups: those with lung involvement and those without lung involvement.

### 2.2. ELISA tests

Peripheral venous blood samples were collected at presentation. The blood samples were centrifuged at 3000 x g for 10 min and the sera were stored at –80 °C. On the evaluation day, the sera were melted at room temperature. When the samples had higher concentrations, they were diluted and measured in duplicate.

The concentrations of TWEAK (Human Tumour Necrosis Factor Related Weak Inducer of Apoptosis, Cat. No. E1820Hu, Bioassay Technology Laboratory, Shangai, China), LT - E4 (Human Human Leukotriene, Cat. No. CSB - E05176h, Cusabio Biotech Co Ltd., Wuhan, China), and Prostaglandin F2α (PGF2α) (Human Prostaglandin F2α, Cat. No. CSB - E10142h, Cusabio Biotech Co Ltd., Wuhan, China) in serum were determined using commercially available Enzyme Linked Immunosorbent Assay (ELISA) kits. The enzymatic reactions were quantified in an automatic microplate photometer. The concentrations of them were determined by comparing the optic density of the samples to the standard curve. All assays were conducted according to the instructions of the manufacturers.

### 2.3. Statistical analysis

Statistical analysis was performed using the Statistical Package for the Social Sciences (SPSS 19.0, Chicago, IL, USA). Quantitative data were expressed as mean ± standard deviation (SD). Normal distributions were tested with the Kolmogorov–Smirnov test with Bonferroni correction. Parametric data were analyzed using the Student’s *t-* test (age, hemoglobin, leukocyte, aspartate aminotransferase (AST), alanine aminotransferase (ALT), platelet, fibrinogen, TWEAK, LTE4, PGF2α). Mann–Whitney U test was performed to compare nonparametric data and to compare the skewed data (neutrophil, lymphocyte, C-reactive protein, and ferritin). Bivariate pearson correlation analysis was used for the linear variables. ROC analysis was performed for TWEAK, LTE4, and PGF2α, and ROC curves for them were drawn. P values less than 0.05 were considered as significant.

## 3. Results

**Figure 1:**
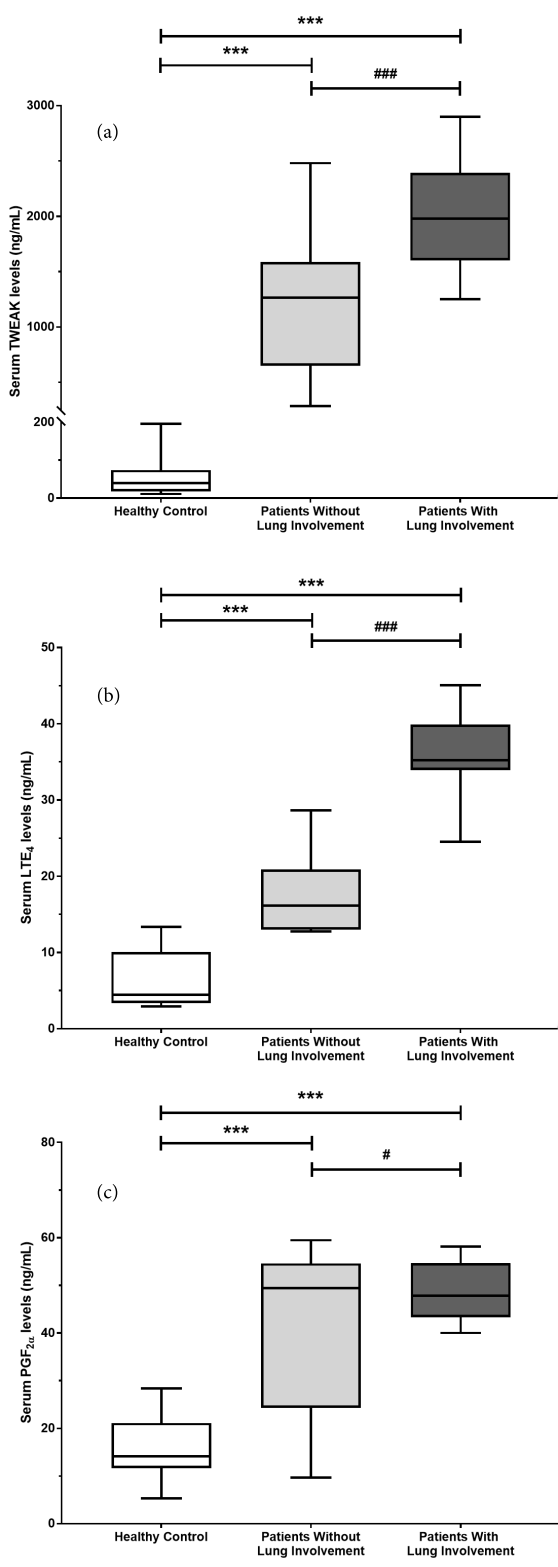
Tumor necrosis factor-like weak inducer of apoptosis (TWEAK) (A), leukotriene (LT) E4 (B), and prostaglandin (PG) F2α (C) values for all groups. Data are represented as mean ± SD. Significance was indicated with asterisk and numerical sign as following ***P < 0.001 versus control and ###P < 0.001, #P < 0.05 versus patients without lung involvement group.

Thirty-five patients with COVID-19 with lung involvement, 25 asymptomatic patients without lung involvement, and 22 healthy volunteers, as the control group, were included in the study. TWEAK (Figure 1A), LTE4 (Figure 1B), and PGF2α (Figure 1C) levels were significantly higher in the group of SARS-CoV-2 infection without lung involvement compared with the healthy control group. In contrast, in the group of COVID-19 with lung involvement, TWEAK, LTE4, and PGF2α levels were significantly higher compared with those without lung involvement and the healthy control group (for all P < 0.001).

Age, aspartate aminotransferase, alanine aminotransferase, fibrinogen, sedimentation, C - reactive protein, and ferritin levels were significantly higher (Table) and lymphocyte levels were significantly lower in COVID-19 patients with lung involvement compared with those without lung involvement.

**Table:** Demographic and laboratory data of study groups.

	Healthy control	Patients without lung involvement	Patients withlung involvement	P value
Sex (female / male)	10 / 9 (53%)	9/16 (36%)	13/22 (37%)	0.573
Age (years)	39.5 ± 10.7	41.2 ± 21.3	64.6 ± 12.5	<0.001
Hemoglobin (mg / dL)	13.87 ± 0.75	14.10 ± 1.20	14.3 ± 2.3	0.938
WBC (mm3)	7758 ± 719	7749 ± 3833	6682 ± 3980	0.594
AST (U / L)	17.03 ± 3.66	24.90 ± 6.50	38.2 ± 19.2	0.016
ALT (U / L)	15.27 ± 2.29	16.6 ± 7.2	30.5 ± 12.8	<0.001
Neutrophil (mm3)	3513 ± 940	4802 ± 3591	4200 ± 2010	0.677
Neutrophil (%)	63.05 ± 7.56	58.5 ± 5.7	68.5 ± 7.4	<0.001
Lymphocyte (mm3)	1458 ± 194.75	1964 ± 984	1305 ± 650	0.07
Lymphocyte (%)	27.84 ± 4.01	27.40 ± 11.50	22.8 ± 11.8	0.350
Platelet (103 / mm3)	249.42 ± 38.82	219 ± 24.0	210 ± 14.0	0.518
Fibrinogen (mg / dL)	238.68 ± 13.58	316 ± 108.0	565 ± 162.0	< 0.001
CRP (mg / L)	1.42 ± 0.50	13.0 ± 12.8	48 ± 30.5	< 0.001
ESR (mm / h)	3.38 ± 1.01	19.70 ± 20.40	82.7 ± 28.7	< 0.001
Ferritin (ng / mL)	123 ± 52	141 ± 128	408 ± 154	< 0.001

WBC: White blood cells, AST: Aspartate transaminase, ALT: Alanine transaminase, CRP: C-reactive protein, ESR: Erythrocyte sedimentation rate

In the study, the age was correlated with hemoglobin (r = –0.464, P < 0.001), lymphocyte (r = –0.337, P < 0.001), erythrocyte sedimentation rate (ESR) (r = 0.652, P = 0.002), C - reactive protein (CRP) (r = 0.380, P < 0.001), and LTE4 (r = 0.378, P = 0.004). LTE4 was correlated with lymphocyte (r = 0.360, P < 0.006), platelet (r = - 336, p < 0.001), CRP (r = 0.362, P < 0.006), fibrinogen (r = 0.456, P < 0.001), TWEAK (r = 0.548, P < 0.001), and PGF2 α (r = 0.644, P < 0.001). PGF2α was correlated with CRP (r = 0.292, P = 0.028) and TWEAK (r = 0.319, P = 0.004).

**Figure 2:**
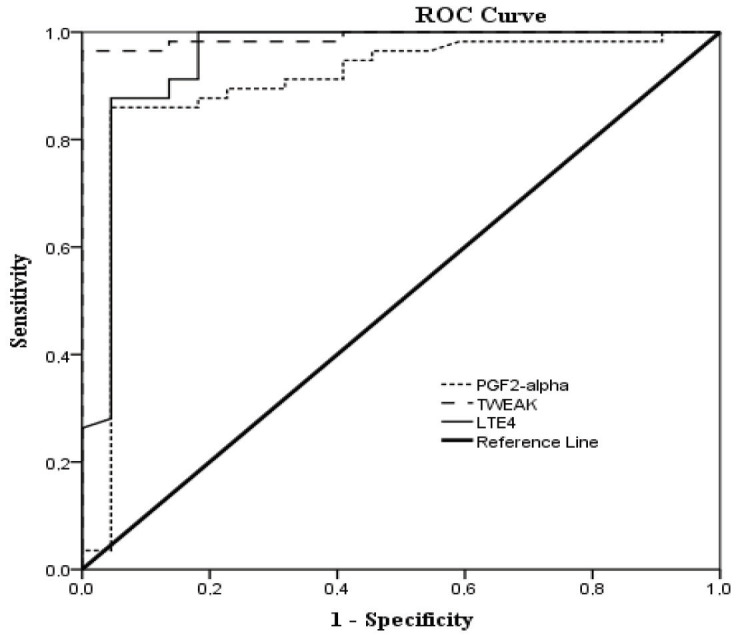
ROC curves of prostaglandin (PG) F2α (AUC: 0.902, p: 0.001), tumor necrosis factor-like weak inducer of apoptosis (TWEAK) (AUC: 0.990, P = 0.001), leukotriene (LT) E4 (AUC: 0.952, P = 0.001) in the healthy control versus the COVID-19.

Receiver Operating Characteristic (ROC) curve analysis was performed to establish the optimal discriminatory threshold to identify patients with COVID-19 from healthy controls. All three markers have the capability of effectively discriminating patients with COVID-19 from HCs. The area under the curve (AUC) of PGF2α, TWEAK, and LTE4 was 0.90 (95% CI 0.810–0.993, P < 0.001), 0.99 (95% CI 0.974–1.000, P < 0.001), and 0.95 (95% CI 0.886–1.000, P < 0.001), respectively (Figure 2). Cut points calculated according to Youden Index.  A level of 27.45 mg/dL (sensitivity 86%, specificity 91%) for PGF2α, 240.85 mg/dL (sensitivity 97%, specificity 100%) for TWEAK, and 13.40 mg/dL (sensitivity 88%, specificity 96%) for LTE4 were found as the optimum cutoff points for defining COVID-19.

## 4. Discussions

In this study, the possible role of TWEAK and LTE4 in SARS-CoV-2 infection, which is considered to cause morbidity and mortality with aberrant immune response rather than its cytopathic effect, was investigated. TWEAK and LTE4 levels were higher in both groups with the disease compared with the healthy control group. Compared with asymptomatic SARS-CoV-2 infection cases, COVID-19 patients with lung involvement were older, and their levels of acute phase reactants and oxidative stress markers (PGF2α) were higher, which is consistent with a more severe clinical presentation of infection. Moreover, the TWEAK level, which has been shown to play an important role in the pathogenesis of autoimmune diseases, and LTE4 levels that play a crucial role in hypersensitivity reactions such as asthma, were higher in COVID-19 cases with lung involvement.

It has been demonstrated that the TWEAK/Fn14 pathway is involved in the pathogenesis of diseases in which the immune system does not have a protective role but is destructive against the host itself [12,14]. TWEAK, Fn14, and RANKL expressions are higher in serum and synovial fluid in RA patients compared with patients with osteoarthritis [20]. In a study by Park et al., it was found that as serum TWEAK levels increased, RA disease activity also increased [21]. These results suggested that blocking the TWEAK-Fn14 pathway could be effective in RA patients. Wisniacki et al. administered TWEAK-blocking monoclonal antibody (BIIB023) to patients with RA and showed that inflammatory cytokines were downregulated, and they have claimed that TWEAK blockers can be effective in diseases for which TWEAK expression has been found to be high [12]. In the present study, we have shown an increased TWEAK expression in COVID-19, as seen in autoimmune diseases. This result suggests that the TWEAK/Fn14 pathway is possibly involved in the immune response to SARS-CoV-2. Therefore, it suggests that there is an aberrant immune response in COVID-19, and antiTWEAK monoclonal antibodies may be an effective treatment method for COVID-19.

An intensive increase in proinflammatory cytokines has been reported in severe clinical presentations and deaths associated with SARS-CoV-2 infection. The resulting cytokine storm leads to cardiovascular collapse, multiorgan failure, and rapid death [2,22]. It has been noted that IL-6 plays the most important role in the pathogenesis of cytokine storm, which develops secondary to SARS-CoV-2 infection [23]. Therefore, tocilizumab is recommended in COVID-19 treatment protocols (Turkish Ministry of Health Guide for COVID-19), and the effectiveness of tocilizumab therapy has also been demonstrated in clinical trials [3,23]. Conversely, in experimental studies, TWEAK/Fn14 activation increases the secretion of many proinflammatory cytokines including IL-6 [10], whereas antiTWEAK antibodies have been shown to decrease the expression of IL-6 and TNFα in animal models [24]. This indicates that the TWEAK/Fn14 pathway may have an important role in excessive proinflammatory cytokine release and development of cytokine storm in SARS-CoV-2 infection.

It is claimed that not only cytokine storm but also eicosanoid storm has a role in the pathogenesis of severe disease in COVID-19 [25]. Massive cell death and cellular debris caused by SARS-CoV-2 stimulate the inflammasome complex and [26] initiate macrophage-derived eicosanoid storm [27]. Proinflammatory bioactive lipid mediators such as prostaglandin and LT fuel local inflammation [28]. Thus, a hyperinflammatory clinical condition without resorption arises, which is resistant to treatments. In the present study, high LTE4 and PGF2α levels found in patients with SARS-CoV-2 infection support the view that hyperinflammation associated with eicosanoid storm is involved in the pathogenesis of aggravation of the disease in COVID-19.

It is claimed that montelukast, a potent cysteinyl leukotriene receptor antagonist, can be a potential therapeutic agent in COVID-19 [29]. Montelukast suppresses leukotriene-mediated inflammatory response by inhibiting the binding of leukotrienes to the receptor [30]. The most important cause of COVID-19 related deaths is ARDS. The pathogenesis of ARDS, manifested by diffuse alveolar damage caused by intense inflammation, has not been fully understood. However, it is known that LTE4 and prostaglandins play an important role [31]. In ARDS patients, leukotriene levels increased by 20 times, and even by 150 times in complicated cases of ARDS in comparison with healthy volunteers [31]. Subsequent studies have also supported the importance of leukotrienes in the pathogenesis of ARDS [17]. Moreover, it has been shown in experimental studies that cysteinyl leukotriene blockers may have favorable effects in the clinical course of ARDS [17]. In the present study, the higher LTE4 and PGF2α levels in COVID-19 patients with lung involvement compared with the asymptomatic group shows the importance of LTE4 and PGF2 in lung inflammation and indicate that montelukast, a leukotriene receptor blocker, can positively contribute to the treatment of cases with SARS-CoV-2 infection associated pneumonia and ARDS.

In conclusion, the immune system can sometimes respond in a hypersensitive manner to infectious agents, just as it does to allergens. In such cases, rather than the direct cytopathic effect of the infectious agent, the aberrant response of the immune system comes to the fore in the clinical presentation, and the destructive effects occur via the immune system. In the present study, the expression of TWEAK/Fn14 pathway and leukotrienes, which have important roles in aberrant immune response reactions, was found to have increased in COVID-19. When an aberrant immune response is triggered in SARS-CoV-2 infection, the probability of treatment success is significantly reduced, and the disease rapidly leads to death. Therefore, TWEAK/Fn14 pathway blockade and cysteinyl receptor blockers may provide a new hope for the treatment of COVID-19.

## Informed Consent

Approval was obtained from the Ethics Committee of Ondokuz Mayıs University, Medical Faculty. The procedures used in this study adhere to the tenets of the Declaration of Helsinki. (OMUKAEK: 2020000278-1)

An informed consent was obtained from all participants prior to their inclusion in the study.
